# Gender differences in the influence of obstructive sleep apnea on optic nerve head circulation

**DOI:** 10.1038/s41598-019-55470-7

**Published:** 2019-12-11

**Authors:** Tomoaki Shiba, Mao Takahashi, Tadashi Matsumoto, Yuichi Hori

**Affiliations:** 1 Department of Ophthalmology, School of Medicine, Toho University, Tokyo, Japan; 2 Cardiovascular Center, Toho University Sakura Medical Center, Chiba, Japan

**Keywords:** Cardiology, Sleep disorders

## Abstract

We investigated gender differences in the optic nerve head (ONH) microcirculation status in association with obstructive sleep apnea (OSA) by using laser speckle flowgraphy (LSFG). We evaluated 150 men (60.5 ± 11.0 yrs) and 45 women (63.0 ± 10.6 yrs) who underwent overnight polysomnography. The mean blur rate (MBR), maximum (Max) MBR, and minimum (Min) MBR were evaluated. The parameters were analyzed separately for the tissues, vessels, and throughout the ONH (All). The apnea hypopnea index (AHI: times/hr), the lowest SpO2%, and the mean SpO2% were calculated as indicators of OSA. We investigated which MBR sections are correlated with OSA parameters separately in the men and women. All MBR sections in the women were significantly positively correlated with the lowest SpO2. In the men, no MBR section was correlated with any OSA parameters. The factors contributing independently to MBR-Tissue were height (β = 0.31) and lowest SpO2 (β = 0.30). The lowest SpO2 in the women was significantly positively correlated with Max MBR-Tissue, Max MBR-All, and Min MBR-All. Our results confirmed a gender difference in characteristics of ONH microcirculation in association with OSA.

## Introduction

An association between arteriosclerotic diseases and obstructive sleep apnea (OSA) was recently identified, and it has long been speculated that OSA may be a risk factor for microangiopathic disorders (e.g., renal disease) and macroangiopathies (e.g., hypertension, coronary artery disease, and cerebrovascular disease)^1–5^. There are numerous reports about relationships between OSA and eye disorders, e.g., open angle glaucoma^6–8^, ischemic optic neuropathy^9^, retinal vein occlusion^10^, and diabetic retinopathy^11–13^. A multi-ethnic cohort study revealed that OSA was associated with narrower retinal arterioles in women but not in men, whereas OSA was associated with incident coronary artery disease in men^14^. Another multicenter cross-sectional study revealed that the associations of OSA severity with retinal microvascular signs may differ by gender^15^. This evidence suggests potential gender differences in the susceptibility to microvascular disease in association with OSA.

Laser speckle flowgraphy (LSFG), a noninvasive quantitative method of determining the ocular blood flow^16,17^, is based on the changes in the speckle pattern of laser light reflected from the fundus of the eye^18^. LSFG is dependent on the movement of erythrocytes in the retina, the choroid, and the optic nerve head (ONH). The mean blur rate (MBR), which is automatically calculated from variations in the degree of blurring, is a quantitative index of the blood flow^19–21^, and measurements of the MBR are highly reproducible^22^. A decreased MBR indicates a reduction in the ocular blood flow of the measurement area^20,21^.

We hypothesized that there are one or more gender differences in the ocular microcirculation affected by OSA, and we speculated that if such differences exist, their clarification could provide clues to the pathophysiology of potential gender differences in the susceptibility to macrovascular and microvascular diseases in association with OSA. We thus conducted the present study using LSFG to investigate gender differences in microvascular characteristics in association with OSA by determining the influence of OSA on the ONH microcirculation of men and women.

## Results

Table 1 summarizes the evaluated parameters of the 150 men and 45 women. The ages of the men (60.5 ± 11.0 yrs) and the women (63.0 ± 10.6, p = 0.17 yrs) did not differ significantly. Among the polysomnography results, the AHI of the men was significantly higher than that of the women (p = 0.01), whereas the mean SpO2 in the women was significantly higher than that in the men (p = 0.02). The lowest SpO2 did not differ significantly between the men and women (p = 0.38). Of the LSFG variables, the MBR-Vessel (p = 0.01) and MBR-All (p = 0.004) in the men were significantly lower than those in the women. Among the 150 men and 45 women, normal-to-mild OSA was present in 24 men (six of whom had an AHI below 5 times/hr) and 14 women (five of whom had an AHI below 5 times/hr); moderate OSA was present in 31 men and 12 women; severe OSA was present in 68 men and 14 women; and very severe OSA was present in 27 men and 5 women (p = 0.13, Yates m × n chi-square test).

**Table 1:** Evaluation parameters of the men and women.

Parameter	Men (n = 150)/Women (n = 45)	p-value
Age, yrs	60.5 ± 11.0/63.0 ± 10.6	0.17^a^
Height, cm	167.7 ± 6.3/153.4 ± 5.3	<0.0001^a^
Weight, kg	71.6 ± 12.9/57.5 ± 10.5	<0.0001^a^
BMI, kg/ m^2^	25.4 ± 3.9/24.4 ± 4.2	<0.0001^a^
Diabetes mellitus, %	39 (26.0)/11 (24.4)	0.83^b^
Hypertension, %	98 (65.3)/21 (46.7)	0.02^b^
Coronary artery disease, %	33 (22.0)/4 (8.9)	0.08^c^
Heart rate, bpm	66.6 ± 9.5/ 73.4 ± 11.8	0.0001^a^
MABP, mmHg	92.1 ± 11.4/87.5 ± 11.1	0.02^a^
Pulse pressure, mmHg	55.4 ± 13.1/57.3 ± 13.7	0.40^a^
Spherical equivalent, D	−0.5 ± 2.5/−0.5 ± 2.8	0.87^d^
IOP, mmHg	12.7 ± 2.9/13.1 ± 3.3	0.45^a^
OPP, mmHg	48.7 ± 7.8/45.2 ± 7.4	0.009^a^
HbA1c, %	6.1 ± 0.9/6.2 ± 1.0	0.59^a^
Red blood cells, ×10^4^/μl	463.2 ± 48.1/436.8 ± 39.1	0.009^a^
Platelets, ×10^3^/μl	21.6 ± 5.5/23.3 ± 8.0	0.11^a^
Cystatin C, mg/l	0.85 ± 0.16/0.75 ± 0.14	0.0003^d^
AHI, times/hr	39.2 ± 22.2/29.3 ± 21.9	0.01^a^
Lowest SpO2, %	81.7 ± 7.6/82.9 ± 8.3	0.38^a^
Mean SpO2, %	94.9 ± 1.7/95.6 ± 1.7	0.02^a^
MBR-Vessel	43.2 ± 7.5/46.4 ± 8.0	0.01^a^
MBR-Tissue	12.6 ± 2.7/13.3 ± 2.7	0.12^a^
MBR-All	23.3 ± 5.1/25.8 ± 5.7	0.004^a^

The data are mean ± SD or number (%). ^a^Unpaired t-test, ^b^2 × 2 chi-square test, ^c^Yates 2 × 2 chi-square test, ^d^Mann-Whitney U-test. AHI: apnea hypopnea index, BMI: body mass index, bpm: beat per minute, D: diopters, HbA1c: glycated hemoglobin A1c, IOP: intra-ocular pressure, MABP: mean arterial blood pressure, MBR: mean blur rate, OPP: ocular perfusion pressure, SpO2: percutaneous oxygen saturation.

The results of the univariate regression analysis between the OSA parameters and the MBR variables in the total cohort and in both genders are shown in Table 2. In the total cohort and in the group of men, none of the MBR variables were significantly correlated with any of the OSA parameters. The MBR-Tissue in the women tended to be correlated with the AHI, but this did not reach significance (r = −0.29, p = 0.05). The lowest SpO2 in the women was significantly positively correlated with the MBR-Vessel (r = 0.42, p = 0.004), the MBR-Tissue (r = 0.32, p = 0.03), and the MBR-All (r = 0.40, p = 0.007).

**Table 2:** Correlation coefficients from the univariate regression analysis between parameters of obstructive sleep apnea and MBR variables of the total cohort and both genders.

Total (n = 195)	MBR-Vessel		MBR-Tissue		MBR-All	
	r	p	r	p	r	p
AHI	−0.06	0.45	−0.07	0.33	−0.07	0.33
Lowest SpO2	0.09	0.24	0.12	0.09	0.14	0.05
Mean SpO2	0.07	0.32	0.08	0.26	0.10	0.17
**Men (n = 150)**	**MBR-Vessel**		**MBR-Tissue**		**MBR-All**	
	**r**	**p**	**r**	**r**	**p**	**r**
AHI	0.04	0.59	0.02	0.81	0.04	0.64
Lowest SpO2	−0.04	0.60	0.05	0.51	0.03	0.72
Mean SpO2	−0.02	0.81	0.04	0.65	0.01	0.89
**Women (n = 45)**	**MBR-Vessel**		**MBR-Tissue**		**MBR-All**	
	**r**	**p**	**r**	**p**	**r**	**p**
AHI	−0.24	0.12	−0.29	0.05	−0.25	0.10
Lowest SpO2	0.42	0.004	0.32	0.03	0.40	0.007
Mean SpO2	0.25	0.10	0.16	0.30	0.23	0.13

Table 3 provides the results of the univariate regression analysis with parameters of OSA and MBR variables with the subjects divided into obese (BMI > 25 kg/m^2^) and non-obese (BMI < 25 kg/m^2^) subgroups in both genders. MBR-All in the obese women was significantly positively correlated with the lowest SpO2 (r = 0.52, p = 0.049), whereas MBR-Vessel in the non-obese women was significantly positively correlated with the mean SpO2 (r = 0.52, p = 0.004).

**Tablee 3:** Univariate regression analysis of parameters of OSA and MBR variables in the patients divided into obese and non-obese subgroups in both genders.

Obese Men (n = 75)	MBR-Vessel		MBR-Tissue		MBR-All	
	r	p	r	p	r	p
AHI	0.05	0.65	0.10	0.38	0.09	0.46
Lowest SpO2	−0.05	0.70	−0.02	0.89	−0.02	0.90
Mean SpO2	0.001	0.99	0.02	0.89	0.03	0.82
**Non Obese Men (n = 75)**	**MBR-Vessel**		**MBR-Tissue**		**MBR-All**	
	**r**	**p**	**r**	**p**	**r**	**p**
AHI	−0.03	0.83	−0.003	0.98	−0.02	0.85
Lowest SpO2	0.04	0.77	0.07	0.54	0.10	0.38
Mean SpO2	0.02	0.84	0.02	0.89	0.01	0.92
**Obese Women (n = 15)**	**MBR-Vessel**		**MBR-Tissue**		**MBR-All**	
	**r**	**p**	**r**	**p**	**r**	**p**
AHI	−0.08	0.79	−0.51	0.06	−0.23	0.41
Lowest SpO2	0.43	0.11	0.47	0.08	0.52	0.049
Mean SpO2	−0.08	0.79	0.24	0.38	0.11	0.71
**Non Obese Women (n = 30)**	**MBR-Vessel**		**MBR-Tissue**		**MBR-All**	
	**r**	**p**	**r**	**p**	**r**	**p**
AHI	−0.23	0.23	−0.25	0.18	−0.13	0.50
Lowest SpO2	0.33	0.07	0.30	0.10	0.24	0.21
Mean SpO2	0.52	0.004	0.12	0.53	0.27	0.15

Table 4 provides the correlation coefficients from the univariate regression analysis for the women between MBR-All, MBR-Vessel, and MBR-All and the clinical parameters. MBR-Vessel was significantly negatively correlated with age (r = −0.34, p = 0.02) and BMI (r = −0.39, p = 0.008) in the women. The MBR-Vessel was significantly correlated only with height (r = 0.33, p = 0.03). The MBR-All was significantly negatively correlated with BMI (r = −0.31, p = 0.03).

**Table 4:** Correlation coefficients from the univariate regression analysis between MBR-Vessel, MBR-Tissue, and MBR-All and clinical parameters in the women.

Objective variable	MBR-Vessel		MBR-Tissue		MBR-All	
Explanatory variables	r	p	r	p	r	p
Age	−0.34	0.02	−0.17	0.26	−0.25	0.09
Height	0.22	0.14	0.33	0.03	0.28	0.07
Weight	−0.28	0.06	0.11	0.47	−0.19	0.22
BMI	−0.39	0.008	−0.01	0.94	−0.31	0.04
Diabetes mellitus	−0.26	0.08	−0.01	0.96	−0.19	0.22
Hypertension	0.12	0.44	−0.20	0.19	−0.11	0.46
Heart rate	−0.21	0.16	−0.16	0.29	−0.11	0.49
MABP	0.19	0.20	0.05	0.74	0.21	0.17
Pulse pressure	−0.04	0.81	0.06	0.68	−0.06	0.71
Spherical equivalent	0.08	0.60	−0.19	0.22	0.08	0.59
IOP	0.01	0.94	−0.03	0.85	−0.04	0.80
OPP	0.19	0.21	0.06	0.69	0.23	0.13
HbA1c	−0.19	0.21	−0.15	0.34	−0.20	0.18
Red blood cells	−0.21	0.18	0.11	0.46	−0.07	0.63
Platelets	0.05	0.74	0.08	0.62	0.18	0.23
Cystatin C	−0.08	0.59	0.13	0.41	0.14	0.35

BMI: body mass index, IOP: intra-ocular pressure, MABP: mean arterial blood pressure, MBR: mean blur rate, OPP: ocular perfusion pressure, HbA1c: glycated hemoglobin A1c, SpO2: percutaneous oxygen saturation.

We next conducted multivariate regression analyses for factors independently contributing to MBR-All, MBR-Vessel, and MBR-All in the women (Table 5). None of the factors were revealed as an independent contributing factor for MBR-Vessel or MBR-All. The factors contributing independently to the MBR-Tissue were height (β = 0.31, p = 0.03) and lowest SpO2 (β = 0.30, p = 0.03).

**Table 5:** Results of the multivariate regression analyses for factors independently contributing to MBR-All, MBR-Vessel, and MBR-All in the women.

Objective variable	MBR-Vessel		MBR-Tissue		MBR-All	
Explanatory variables	β	p	β	p	β	p
Age	−0.29	0.047				
Height			0.31	0.03		
BMI	−0.30	0.08			−0.13	0.49
Lowest SpO2	0.16	0.36	0.30	0.03	0.33	0.06

R = 0.53, p = 0.01; R = 0.44, p = 0.001; R = 041, p = 0.02.

The R values of each multivariate regression analysis are shown.

The results for the men revealed that only the spherical equivalent was correlated with MBR-Vessel by a univariate regression analysis (r = 0.30, p = 0,0008). The red blood cell count (β = −0.30, p = 0.0006) and hypertension (β = −0.26, p = 0.003) were identified as contributing factors for MBR-Tissue. No factors were correlated with MBR-All in the men.

We obtained the correlation coefficients from the univariate regression analysis between Max MBR-Tissue, Min MBR-Tissue, and MBR-All and the lowest SpO2 (Table 6). The lowest SpO2 in the women was significantly positively associated with Max MBR-Tissue (r = 0.30, p = 0.046), Max MBR-All (r = 0.39, p = 0.008), and Min MBR-All (r = 0.38, p = 0.01) and tended to be associated with Min MBR-Tissue (r = 0.28, p = 0.06), although this tendency was not significant.

**Table 6:** Univariate regression analysis of the associations between lowest SpO2 and Max MBR-Tissue, Min MBR-Tissue, and Min MBR-All in the women.

Explanatory variable	r	p
Max MBR-Tissue	0.30	0.046
Min MBR-Tissue	0.28	0.06
Max MBR-All	0.39	0.008
Min MBR-All	0.38	0.01

Objective variable: Lowest SpO2.

## Discussion

Aging has been identified as a factor contributing to a decrease in the MBR in the ONH^22–24^. It was also reported that the intima-media thickening revealed by ultrasonography reflects a decrease in all of the sections of the MBR in the ONH of individuals with diabetes mellitus^25^. The progression of a visual field defect in patients with open angle glaucoma was reported to reflect a decrease in all sections of the MBR^26^. Our previous investigation clarified that (1) the MBR-Tissue value was significantly lower in patients with metabolic syndrome compared to patients without metabolic syndrome, and (2) the overlap of the metabolic syndrome components causes decreases in the MBR-Tissue and MBR-All in the ONH^27^. From these reports, it is clear that a low MBR value in the ONH reflects an unfavorable status of an individual’s systemic and/or ophthalmic condition.

Several meta-analyses revealed that OSA is a risk factor for the onset of open angle glaucoma^28–30^. OSA is also well known to have a strong association with metabolic syndrome. It was reported that 50–60% of individuals with metabolic syndrome have OSA^31,32^. A multi-ethnic cohort study revealed that OSA was associated with narrower retinal arterioles in women, but not in men^14^. This finding suggested potential gender differences in the susceptibility to microvascular diseases in association with OSA. We hypothesized that (1) the relationships among OSA, metabolic syndrome, and open angle glaucoma can be explained by the ocular microcirculation, and (2) the influence of OSA on microcirculation is stronger in women than men. The precise influence of OSA on the ONH microcirculation has been unclear, and we thus performed the present study to clarify the relationships between OSA and the ONH microcirculation by using LSFG, separating the patient cohort into the men and women with various systemic statuses.

The polysomnography results demonstrated that the AHI in the men was significantly higher than that in the women, and the mean SpO2 during sleep in the men was significantly lower than that in the women. The lowest SpO2 values did not show a significant difference between the genders (Table 1).

The incidence of OSA is well known to be higher among men than women^2^, as has been reported in Japanese^33^. However, we observed no significant difference between the genders in the distribution of the severity of OSA classifications. Our LSFG measurements revealed that the MBR-Tissue and MBR-All in the men were significantly lower than those in the women. Several reports regarding a survey of healthy subjects noted that gender differences affected the MBR in the ONH^24,34^. It has been reported that all sections of the MBR^24^, the MBR-vessel, and the MBR-All ^34^ of men were significantly lower than those of women. The LSFG measurements in our previous cross-sectional study of patients with some arteriosclerotic complications were similar to the measurements in these prior reports^24,34^.

We obtained correlation coefficients from the univariate regression analysis between OSA parameters and all sections of the MBR of the total patient cohort and both genders (Table 2), and there was no significant correlation between any of the OSA parameters and any of the MBR sections in our evaluations of the total cohort and the men. Our results thus indicate that the lowest SpO2 is associated with all sections of the MBR in women, but not in men. Because the OSA severity grades that we used were not significantly different between the genders, our findings suggest that gender differences are present in the susceptibility to ONH microcirculation abnormality in association with OSA.

Considering MBR-Tissue in the women in this study, the AHI tended to be associated with MBR-Tissue; however, the lowest SpO2 was identified as a factor contributing to MBR-Tissue. There is a possibility that the number of sleep apnea events represented by the AHI is also important, but severe hypoxia and reoxygenation represented by the lowest SpO2 might cause more serious damage to microvessels via an ischemia and reperfusion injury. In a study of diabetic retinopathy, the lowest SpO2 (among OSA parameters) was identified as the factor contributing to the progression of proliferative diabetic retinopathy^12,35,36^. The lowest SpO2 has also been identified as a risk factor for cardiovascular disease and cerebral infarction in individuals with diabetes mellitus^35,36^. In light of the above-mentioned findings, we speculate that among OSA parameters, the lowest SpO2 may be an important factor in the development of OSA-related macrovascular and/or microvascular disease.

Next, to clarify whether obesity affects the association between parameters of OSA and MBR, we conducted a univariate regression analysis with parameters of OSA and MBR variables in the subjects divided into obese and non-obese subjects in both genders (Table 3). The MBR-All in the obese women was significantly positively correlated with the lowest SpO2, whereas the MBR-Vessel in the non-obese women was significantly positively correlated with the mean SpO2. These results suggested that the influence of OSA on the ONH microcirculation may differ between obese and non-obese women. However, further detailed studies with greater numbers of subjects are needed to test our present findings.

Because there was no significant correlation between any of the OSA parameters and any of the MBR sections in our evaluations of the total cohort and the men, we conducted univariate and multivariate regression analyses to identify any factors that independently contribute to MBR-All, MBR-Vessel, and MBR-All in the women (Tables 4, 5). No single factor was revealed as an independent contributing factor for MBR-Vessel and All, but height and the lowest SpO2 were identified as factors that independently contributed to MBR-Tissue in the women. A high height value was related to increased MBR-Tissue in the women in this study, and a low value of lowest SpO2 reflected low values of MBR-Tissue in the women but not in the men.

It has been speculated that MBR-Tissue is stable regardless of the subjects’ age or gender and may be a quantitative, clinically useful way of identifying circulatory disturbances in ocular diseases^34^. Our novel finding of relationship between OSA and ONH microcirculation may thus support our hypothesis. In any case, our study revealed relationships between the ONH microcirculation and OSA, and it confirmed that a low value of lowest SpO2 leads to decreases in MBR-Tissue independently in women, but not in men. However, in the men, the spherical equivalent, the red blood cell count, and the incidence of hypertension were contributing factors for MBR-Vessel and MBR-All. Our observation that the incidence of hypertension leads to a decrease in the MBR-Tissue in men is a novel finding.

Several research groups have investigated the relationship between ocular circulation and OSA parameters by using the ophthalmic artery resistivity index, the central retinal artery resistivity index, and pulsatile ocular blood flow measurements; no correlation was found between the ocular blood flow and OSA^37,38^. The resistivity index of retinal arterioles and pulsatile ocular blood flow measurements are calculated from the variations of the blood flow during the systolic and diastolic periods^37,38^. We obtained correlation coefficients from the univariate regression analysis between the Max, Min MBR-Tissue, and -All and the lowest SpO2 in the women (Table 6), and the results clarified that the lowest SpO2 influenced both the Max-MBR and the Min-MBR in the ONH. This result indicates that a reduction of the lowest SpO2 causes a decrease in both the Max-MBR and Min-MBR in the ONH (especially the tissue-area) microcirculation without an expansion of the variation of the MBR of the ONH over the cardiac cycle. Therefore, our pulse wave analysis parameters did not show significant correlations with the lowest SpO2 (data not shown).

We used more parameters of OSA in this study and analyzed the data of the two genders separately. As a result, we were able to clarify the relationship between OSA and ocular microcirculation for the first time. A power analysis (using the Correlation: Point-biserial model) by G*Power software (ver. 3.1.3; developed by Franz Faul, Kiel University, Kiel, Germany) showed that a total sample size of 40 is needed for an effect size of 0.85, an error probability (a) of 0.05, and the power (1 – b) of 0.85. Our total subject number is thus large enough for an ideal sample size in both genders.

This study has some major limitations. First, many of the patients had been receiving treatment for hypertension, diabetes mellitus, or other diseases. Because this was a cross-sectional study, we did not evaluate the effects of the treatments for these conditions on the ONH microcirculation as primary endpoints. Second, we did not evaluate the effects of therapy for OSA. The influence of treatments for hypertension, diabetes mellitus, or other diseases including OSA on the ONH microcirculation was also not examined in this study. Finally, polysomnography records many sleep parameters, including the sleep stages. In particular, we did not calculate the cumulative percentage of the period of time at which the SpO2 was below 90%. Further detailed studies using more sleep parameters determined by polysomnography are necessary to address the above-described study limitations.

In conclusion, we observed that a low value of the lowest SpO2 led to low values of MBR-Tissue in the women, but not in the men. Our findings may provide clues to the pathophysiology of potential gender differences in the susceptibility to macrovascular and microvascular diseases in association with OSA.

## Patients and Methods

### Study design

The Ethics Committee of the Toho University Sakura Medical Center approved this study (No. 2010-012), which was prospective and cross-sectional in nature, and all patients provided informed consent to participate in accord with the tenets of the Declaration of Helsinki. This study was registered in the UMIN (ID: UMIN000038230).

### Patients

We studied 236 consecutive patients who had undergone overnight polysomnography at the Department of Cardiovascular Center of the Toho University Sakura Medical Center between April 1, 2007 and the end of December 2012. All patients were Japanese and were referred to the Department of Cardiovascular Center of the Toho University Sakura Medical Center due to suspected sleep apnea syndrome. Patients were excluded if they had central sleep apnea, arrhythmia such as atrial fibrillation, glaucoma, uveitis, optic neuropathy, vitreous or retinal disease, or retinal or choroidal vascular disease, or if they had undergone a previous intraocular surgery. A final total of 195 patients (men = 150, women = 45) met the study criteria.

Blood pressure measurements and LSFG were performed after the patient rested for 10 min in a quiet, air-conditioned room with the temperature maintained at 24 °C. The patients did not fast, but all patients abstained from smoking, alcohol, and caffeine for ≥24 hr prior to the measurements, as described^39^. LSFG, blood pressure, and ophthalmic evaluations were made between 3:00 and 4:00 p.m., before a meal.

### Overnight polysomnography

We performed an overnight sleep study using a computerized polysomnogram system (Alice 5^®^ Diagnostic Sleep System, Hollywood Medical Supply, Hollywood, FL, USA). Recordings were manually scored according to standard criteria^40^. All of the patients underwent overnight polysomnography performed over a minimum of 6 hr in a quiet private room at the Toho University Sakura Medical Center. Electroencephalography, submental electromyography, electro-oculography, nasal and oral airflows measured using thermistors, and pulse oximetry measurements were recorded using a standard technique. The apnea hypopnea index (AHI: times/hr), the lowest percutaneous oxygen saturation during sleep (lowest SpO2: %), and the average SpO2 during sleep (mean SpO2: %) were calculated and used as indicators of the items of OSA.

An apneic event was defined as the cessation of airflow for ≥10 sec with effort to breathe. A hypoapneic event was defined as a minimal 30% reduction in thoracoabdominal movement or airflow compared with the baseline value, lasting ≥10 sec with ≥4% oxygen desaturation^40–42^.

The severity of OSA was graded according to the following AHI values: normal to mild, <15; moderate, ≥15 to <30; severe, ≥30 to <60; and very severe, ≥60^43^.

### Measurements of systemic and laboratory parameters

The following values were used as systemic parameters: age (years), height (cm), weight (kg), body mass index (BMI: kg/m^2^), heart rate (beats per minute: bpm), mean arterial blood pressure (MABP, mmHg) calculated from diastolic blood pressure + (systolic blood pressure − diastolic blood pressure)/3, and pulse pressure (mmHg).

The laboratory profile of each patient was comprised of the determination of the glycated hemoglobin A1c (HbA1c), the red blood cell count (×10^4^/μl), the platelet count (×10^3^/μl), and the cystatin C (mg/l) from fasting morning blood samples. HbA1c measurements are expressed based on the National Glycohemoglobin Standardization Program scale.

### The patients’ medical histories

Each patient’s history of diabetes mellitus, hypertension, and coronary artery disease was checked. A history of coronary artery disease was confirmed using the patient’s medical records. Hypertension was defined as systolic blood pressure >140 mmHg or diastolic blood pressure >90 mmHg. Hypertension was also diagnosed in patients using antihypertensive drugs. Diabetes mellitus was defined as a fasting blood glucose level > 126 mg/dl, HbA1c > 6.5%, or both. Diabetes mellitus was also defined as the use of a hypoglycemic agent.

### LSFG measurements

**Figure 1:**
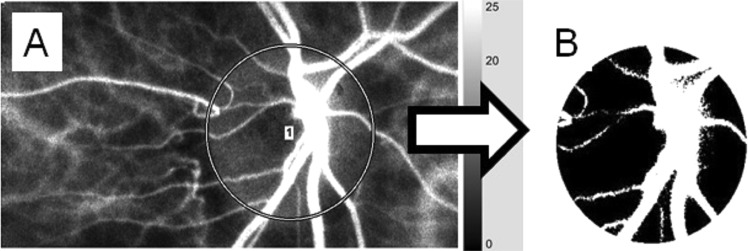
Analysis of the MBR values in the ONH circulation using LSFG. The gray-scale map of the total measurement area is shown. (**A)** The *circle* surrounds the area of the ONH. (**B)** The software separates out the retinal vessels by using an automated definitive threshold throughout the ONH, within the ONH vessel (shown in *white*), and within the ONH tissue (*black*).

**Figure 2:**
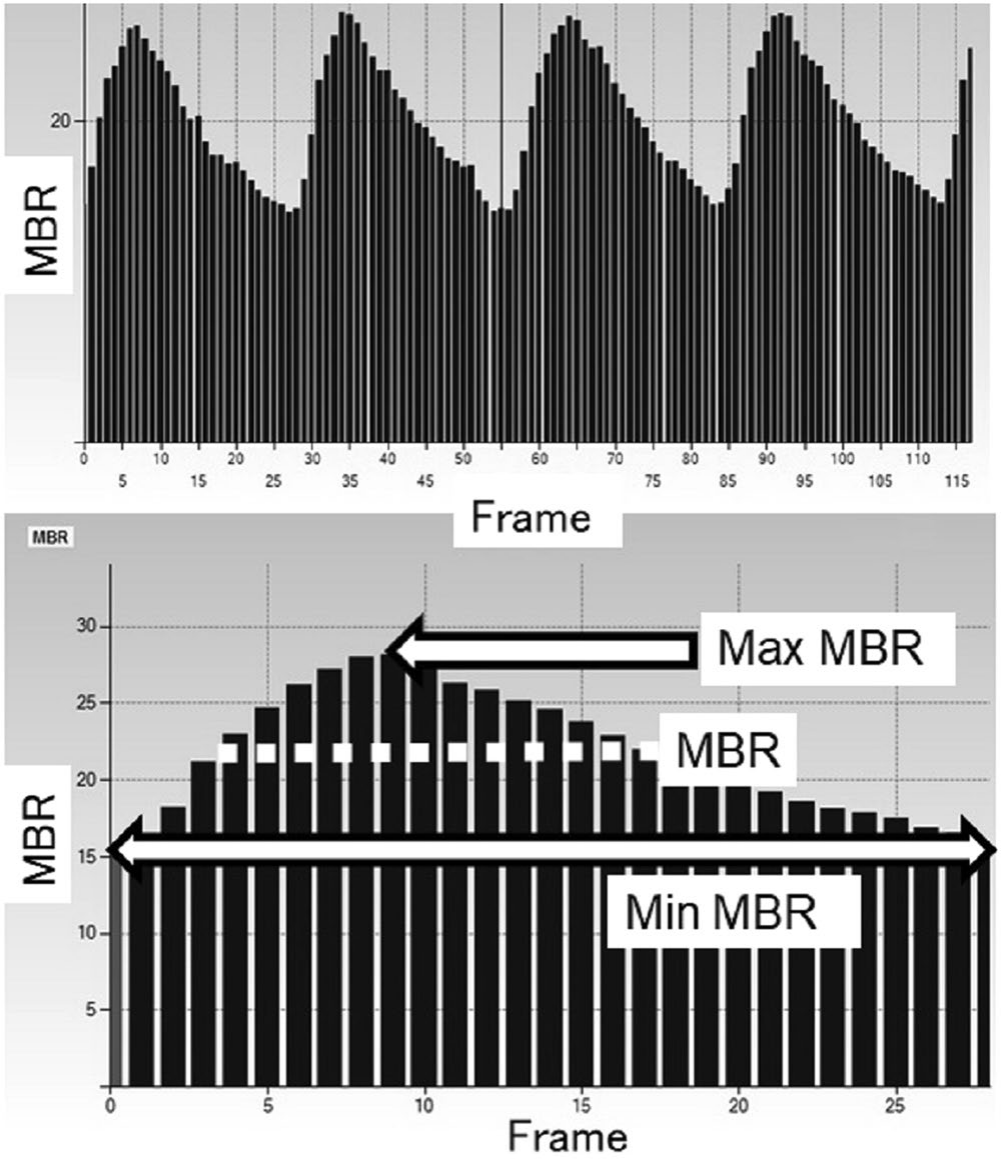
Variation of the MBR, which is tuned to the cardiac cycle for 4 sec. *Upper panel:* The total number of frames was 118. *Lower panel:* The software calculates the normalization of one pulse. MBR, Max-MBR, and Min-MBR in the ONH-Vessel, ONH-Tissue, and whole of the ONH (-All) are calculated on this screen. MBR: mean blur rate. Max-MBR: the maximum MBR value in a heartbeat. Min-MBR: The minimum value of MBR variation in a heartbeat (max. value of steady blood flow).

LSFG images were obtained by an LSFG-NAVI™ system (Softcare Co., Fukuoka, Japan), and the maximum, minimum, and average MBR values were calculated by LSFG Analyzer software (ver. 3.0.47, Softcare). The details of the determination of the LSFG measurements from fundus images were as described^19,44,45^. Briefly, for the evaluation of the patient’s ONH circulation, a circle surrounding the ONH was set on the screen of a personal computer (Fig. 1A). The software separated out the vessels using the automated definitive threshold (Fig. 1B). Within a 4-sec period tuned to the cardiac cycle, 118 MBR images (118 frames) were recorded from the circled area. The analysis of the screen (which was normalized to one pulse automatically) is then displayed (Fig. 2, lower panel), and the analyses of the parameters were made on this screen.

The MBR, which was a main evaluation item, was determined based on the mean quantity of MBR during one normalized cardiac cycle. We also analyzed the maximum MBR (Max-MBR) determined by the peak of MBR during one normalized cardiac cycle (Fig. 2, upper arrow in the lower panel) and the minimum MBR (Min-MBR) determined by the base level of the variation of the MBR during one normalized cardiac cycle (Fig. 2, lower arrow in the lower panel). Each MBR value was analyzed respectively in the ONH tissue (Tissue), in the vessels of the ONH (Vessel), and throughout the ONH (All). All of the measurements were taken with the patient in the seated position, and the patient’s pupils were dilated with 0.5% tropicamide eye drops. Only data from the right eye were used for the analyses.

### Evaluation of other ocular parameters

The following parameters of the patients’ right eyes were measured: the spherical equivalent (diopters; D) assessed with a Tonoref 2™ system (Nidek, Aichi, Japan), intraocular pressure (IOP; mmHg) measured by applanation tonometry, and the ocular perfusion pressure (OPP; mmHg). The OPP was defined as: (2/3 MABP) – IOP. All patients underwent an ophthalmologic examination using slit-lamp biomicroscopy to ensure that they did not have any ocular or systemic conditions that would render them ineligible for analysis.

### Statistical analyses

Data for the continuous variables are presented as the mean ± SD. The unpaired t-test, 2 × 2 chi-square test, Yates 2 × 2 and m × n chi-square tests, and Mann-Whitney U-test were used for the comparison of patient parameters between the genders. We used the unpaired t-test for parametric parameters and the Mann-Whitney U-test for non-parametric parameters. We performed a univariate regression analysis (obtaining Pearson’s correlation coefficients) to determine which section(s) of the MBR in the ONH are significantly correlated with OSA parameters, dividing the patients into the men and the women, and the obese patients (BMI ≥ 25 kg/m^2^) and the non-obese patients (BMI < 25 kg/m^2^). We then conducted univariate and multivariate regression analyses to determine the independent factors for the MBRs in the ONH that are significantly correlated with OSA parameters. Finally, we performed a univariate regression analysis to determine which of the MBR parameters among Max-MBR, Min-MBR, and the mean quantity of MBR are most closely correlated with OSA parameters. P-values < 0.05 were accepted as significant. The Stat View program ver. 5.0 (SAS, Cary, NC) was used for the statistical analyses.
